# Functional Threshold Power Field Test Exceeds Laboratory Performance in Junior Road Cyclists

**DOI:** 10.1519/JSC.0000000000004471

**Published:** 2023-02-02

**Authors:** Giovanni Vinetti, Huber Rossi, Paolo Bruseghini, Marco Corti, Guido Ferretti, Simone Piva, Anna Taboni, Nazzareno Fagoni

**Affiliations:** 1 Department of Molecular and Translational Medicine, University of Brescia, Brescia, Italy;; 2 Institute of Mountain Emergency Medicine, Eurac Research, Bolzano, Italy;; 3 Marathon Sport Medical Center, Brescia, Italy;; 4 Department of Human Sciences and Promotion of the Quality of Life, University San Raffaele Roma, Rome, Italy; and; 5 Department of Medical and Surgical Specialties, Radiological Sciences and Public Health, University of Brescia, Brescia, Italy

**Keywords:** adolescent, critical power, functional threshold power, power meter, training, uphill

## Abstract

Vinetti, G, Rossi, H, Bruseghini, P, Corti, M, Ferretti, G, Piva, S, Taboni, A, and Fagoni, N. The functional threshold power field test exceeds laboratory performance in junior road cyclists. *J Strength Cond Res* 37(9): 1815–1820, 2023—The functional threshold power (FTP) field test is appealing for junior cyclists, but it was never investigated in this age category, and even in adults, there are few data on FTP collected in field conditions. Nine male junior road cyclists (16.9 ± 0.8 years) performed laboratory determination of maximal aerobic power (MAP), 4-mM lactate threshold (P_4mM_), critical power (CP), and the curvature constant (*W*′), plus a field determination of FTP as 95% of the average power output during a 20-minute time trial in an uphill road. The level of significance was set at *p* < 0.05. Outdoor FTP (269 ± 34 W) was significantly higher than CP (236 ± 24 W) and P_4mM_ (233 ± 23 W). The $\dot{V}O_{2\text{peak}}$ of the field FTP test (66.9 ± 4.4 ml·kg^−1^·min^−1^) was significantly higher than the $\dot{V}O_{2\text{peak}}$ assessed in the laboratory (62.7 ± 3.7 ml·kg^−1^·min^−1^). Functional threshold power was correlated, in descending order, with MAP (*r* = 0.95), P_4mM_ (*r* = 0.94), outdoor and indoor $\dot{V}O_{2\text{peak}}$ (*r* = 0.93 and 0.93, respectively), CP (*r* = 0.84), and *W*′ (*r* = 0.66). It follows that in junior road cyclists, the FTP field test was feasible and related primarily to aerobic endurance parameters and secondarily, but notably, to *W*′. However, the FTP field test significantly exceeded all laboratory performance tests. When translating laboratory results to outdoor uphill conditions, coaches and sport scientists should consider this discrepancy, which may be particularly enhanced in this cycling age category.

## Introduction

Performance in long-lasting cycling races can be evaluated with several laboratory-derived and field-derived parameters (18,30). Among them, the functional threshold power (FTP) has been defined as 95% of the average power output (PO) during a 20-minute time trial (TT) in the field measured with a power meter and has been proposed as a surrogate of the maximal PO sustainable for 1 hour (1). Despite its widespread use in professional and amateur cyclists, there is incomplete agreement on the relationships between FTP and traditional exercise intensity boundaries (21). Moreover, although it was intended to be a field test, studies on the physiological underpinnings of FTP were mostly confined to the laboratory setting, where mixed agreement was found with a 60-minute TT (3,20,23), as well as the ventilatory compensation point (2,33), the individual anaerobic threshold (3,20), the Dmax lactate threshold (23,32,35), the 4 mM (16,32) lactate threshold (P_4mM_), the maximal lactate steady state (4,15,19), and the critical power (CP) (17,24,27), with most studies refuting interchangeability. The 20-minute TT naturally evokes the concept of PO–duration (T_lim_) relationship (18), its simplest hyperbolic form being *T*_lim_ = *W*′/(PO–CP), where the curvature constant *W*′ (the amount of work that can be performed above CP) interestingly resulted unrelated to FTP (27). The need of further studies assessing FTP in outdoor conditions was recently highlighted (21), since only 2 studies have investigated the outdoor FTP test to date (both with the modified protocol of 90% of the average PO of an 8-minute TT): 1 (11) reported an FTP not significantly different from P_4mM_ and the other (31) an FTP greater but significantly related to P_4mM_. However, these field results should be taken with caution because of the different bicycles used in the laboratory and field testing (11) or the heterogeneous models of power meters used (31).

The simplicity of the outdoor 20-minute TT could make it also appealing for testing and training of junior road cyclists, but, to the best of our knowledge, it has never been investigated in this age category. Thus, this study aimed to assess the FTP test in field conditions in junior road cyclists and to compare it with laboratory-derived parameters.

## Methods

### Experimental Approach to the Problem

A junior road cycling team performed (a) an indoor incremental test for the determination of P_4mM_ and the maximal aerobic power (MAP) (metabolic, $\dot{V}O_{2\text{peak}}$ and mechanical, MAP), (b) 4 indoor constant-power trials to estimate CP and *W*′, and (c) an outdoor 20-minute TT to calculate FTP. All tests were conducted at least 24 hours apart and concluded within 15 days.

### Subjects

Nine male junior road cyclists (16.9 ± 0.8 years, range 16.0-18.0, 174 ± 5 cm, 65.7 ± 7.6 kg, body fat 9.5 ± 2.2% (8)) were enrolled and completed the study. They were informed about the aims, the procedures, and risks associated with the tests, and they (or their legal representatives if minors) signed a written informed consent form. This study conformed to the Declaration of Helsinki and was approved by the University of Brescia Institutional Review Board. They were asked to avoid heavy exercise and have proper hydration in the 24 hours before each experiment, to have a light meal without coffee intake 2–4 hours before the tests, and to maintain the training workload constant throughout the study period.

### Procedures

In all tests, subjects were on their own road bicycle equipped with a crank-based power meter (Power2max, Type S, Chemnitz, Germany), wearing a portable metabolic cart (K5, COSMED, Rome, Italy) and a heart rate (HR) monitor (HRM-Dual, Garmin, Olathe, KS). The 30-second rolling average of oxygen consumption ($\dot{V}O_{2}$) was measured every 10 seconds at the mouth with dynamic micro mixing chamber technology. The gas analyzers (a galvanic fuel cell O_2_ sensor and an infrared CO_2_ meter) were calibrated with ambient air, with the use of a CO_2_ scrubber sodium and calcium hydroxides and with a mixture of known gases (O_2_ 16%, CO_2_ 5%, and N_2_ as balance) and the turbine flowmeter by means of a 3-L syringe as per manufacturer's guidelines. Capillary blood lactate concentration ([La^−^]) was assessed by an electroenzymatic method (Arkray, Lactate Pro 2, Kyoto, Japan) in 0.3 μL samples taken from the earlobe.

Indoor tests were performed in a sports medicine laboratory (Marathon Sport Medical Center, Brescia, Italy) between 2 and 6 pm. Ambient temperature and relative humidity were 20.2 ± 1.2 °C and 52.5 ± 4.6%, respectively. At the first visit, body mass, height, and skinfold thickness (8) were recorded. Then, subjects performed an incremental test against an electronically controlled magnetic brake mounted in place of the rear wheel of the bicycle (Jarvis Magnetic Days, ORF, Arezzo, Italy). Magnetic resistance was electronically regulated to keep PO independent of pedal cadence, starting from 100 W and then increasing by 50 W every 4 minutes until exhaustion. To be consistent with field conditions only the PO measured by the Power2max power meter was used for this, and all the subsequent tests. [La^−^] was assessed at the end of each step and at minute 1, 3, and 5 of recovery. P_4mM_ was assessed by linear interpolation between PO and [La^−^] (7), whereas MAP by linear extrapolation between PO and steady-state $\dot{V}O_{2}$ (60-second average) up to $\dot{V}O_{2\text{peak}}$, which was determined as the highest 30-second average. Gross efficiency for high-intensity exercise in the laboratory setting was calculated as the ratio between the PO and steady-state $\dot{V}O_{2}$ of the first step above P_4mM_.

On separate days, subjects returned to the laboratory to perform 4 constant-power trials to exhaustion. Power output was selected based on MAP (38): the first 3 trials were at 90, 100, and 110% of MAP, administered in a random order; the last was either 85 or 95% of MAP with the aim to have the longer T_lim_ between 12 and 20 minutes (22). Trials were preceded by a standardized warm-up (20 minutes from 50 to 60% of MAP, then 3 × 60 second sprints up to 100% of MAP interspersed by 60 seconds of rest), 5 minutes of rest for instrumentation, and 60 seconds at 50% of MAP after which the trial's PO was suddenly applied. Standardized verbal encouragement was provided, and T_lim_ was retained when pedal cadence remained below 70 min^−1^ for more than 10 seconds despite warning. [La^−^] was assessed after warm-up and at minute 1, 3, and 5 of recovery. The power-duration relationship was analyzed with the 2-parameter hyperbolic model (28).

**Figure 1.**
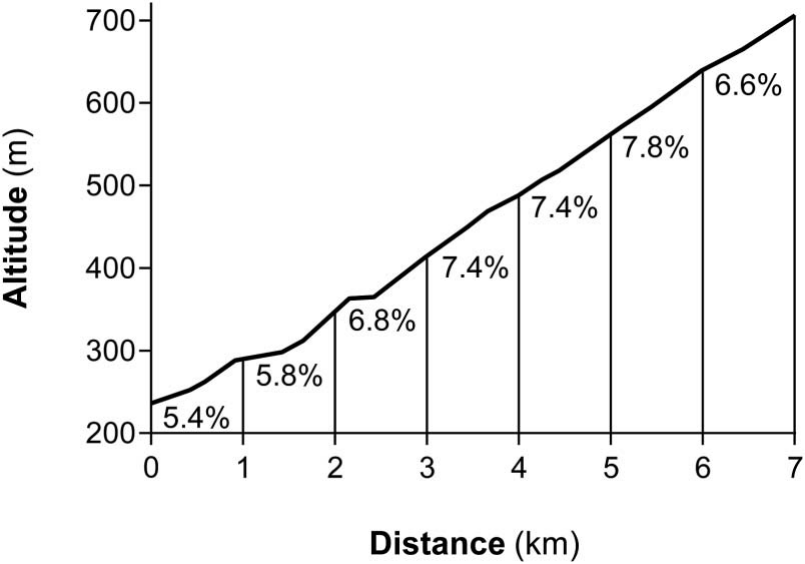
Altimetric profile of the ascent. Labels refer to average grade of each kilometer.

The 20-minute TT was performed in an outdoor uphill road (Monte Maddalena, Brescia, Italy) with an average gradient of 6.7% (Figure 1), on another separate day between 2 and 5 pm. Ambient temperature and relative humidity were 10.1 ± 2.9 °C and 65.2 ± 9.2%, respectively. The TT was preceded by the same standardized stationary warm-up as described above and 5 minutes of rest for instrumentation. [La^−^] was assessed after warm-up and at minute 1, 3, and 5 of recovery. Functional threshold power was calculated by multiplying TT average PO by 0.95 (1). Gross efficiency for the FTP test was calculated as the ratio between the average PO and the average $\dot{V}O_{2}$ of the last 15 minutes of the TT.

### Statistical Analyses

Variables and parameters were compared by means of Bland-Altman plots and reduced major axis linear regression analysis. Values are reported as mean ± standard deviation. The power-duration relationship was fitted by means of geometric mean nonlinear regression method to account for the presence of heteroscedastic random error both in T_lim_ and PO for biological and technological reasons, respectively (37,39). Repeated measures one-way analysis of variance (ANOVA) was used to compare variables between constant-power trials, and the Tukey post hoc test was performed to locate significant difference. The effect size was determined by Cohen's *d* and classified with the Hopkins criteria: 0–0.2 trivial, 0.2–0.6 small, 0.6–1.2 moderate, 1.2–2.0 large, and >2.0 very large (14).The level of significance was set at *p* < 0.05 The statistical package SPSS (Version 23.00, IBM Corp., Armonk, NY) was used.

## Results

### Laboratory Tests

**Figure 2.**
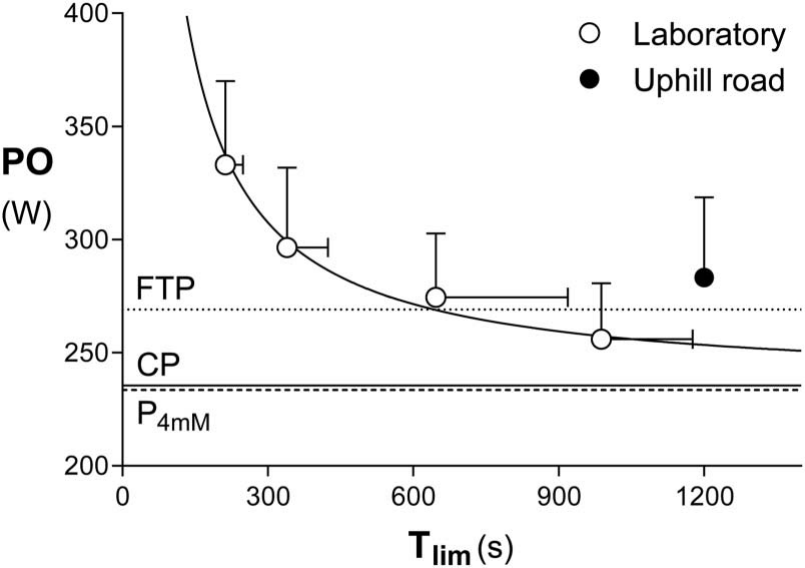
Average power–duration data from the laboratory constant-power trials (open dots) and the field 20-minute time trial (filled dot) and derived parameters: CP (continuous line), FTP (dotted line), P_4mM_ (dashed line). Error bars show standard deviation. CP = critical power; FTP = functional threshold power; P_4mM_ = PO at the 4-mM blood lactate concentration; PO = power output; T_lim_ = duration.

A graphical summary of laboratory and field tests is presented in Figure 2**.** Incremental test peak values of PO, HR, and [La^−^] were 309 ± 30 W, 198 ± 5 beats·min^−1^, and 11.0 ± 1.7 mM, respectively. $\dot{V}O_{2\text{peak}}$ was 4.1 L/min (62.7 ± 3.7 ml·kg^−1^·min^−1^), and extrapolated MAP was 276 ± 28 W. Peak $\dot{V}O_{2}$ and average pedal cadence were constant across all laboratory tests (*p* = 0.48 and 0.50, respectively). There was a significant effect of the laboratory test on peak HR and [La^−^] (*p* = 0.02 and 0.002, respectively). Peak HR of the 3 shorter constant-power trials was moderately lower than the incremental test (*d* = 0.8–1.2), and peak [La^−^] of the 2 longest constant-power trials was largely lower than the incremental test and the shorter constant-power trial (*d* = 1.6 for both). Post–warm-up [La^−^] was 1.6 ± 0.7 mM. Critical power and *W*′ estimates were 236 ± 24 W and 20.7 ± 4.7 kJ (*SE* = 11 ± 9 W and 5.2 ± 5.6 kJ, respectively). P_4mM_ was 233 ± 23 W, not significantly different from CP (*p* = 0.66, *d* = 0.1) and in fair agreement with it (bias 2 ± 13 W, 95% limits of agreement −28 to +24 W, *r* = 0.84). Critical power and P_4mM_ were in good correlation with MAP (*r* = 0.82 and 0.94, respectively) and with $\dot{V}O_{2\text{peak}}$ (*r* = 0.90 for both).

### Functional Threshold Power Field Test

**Figure 3.**
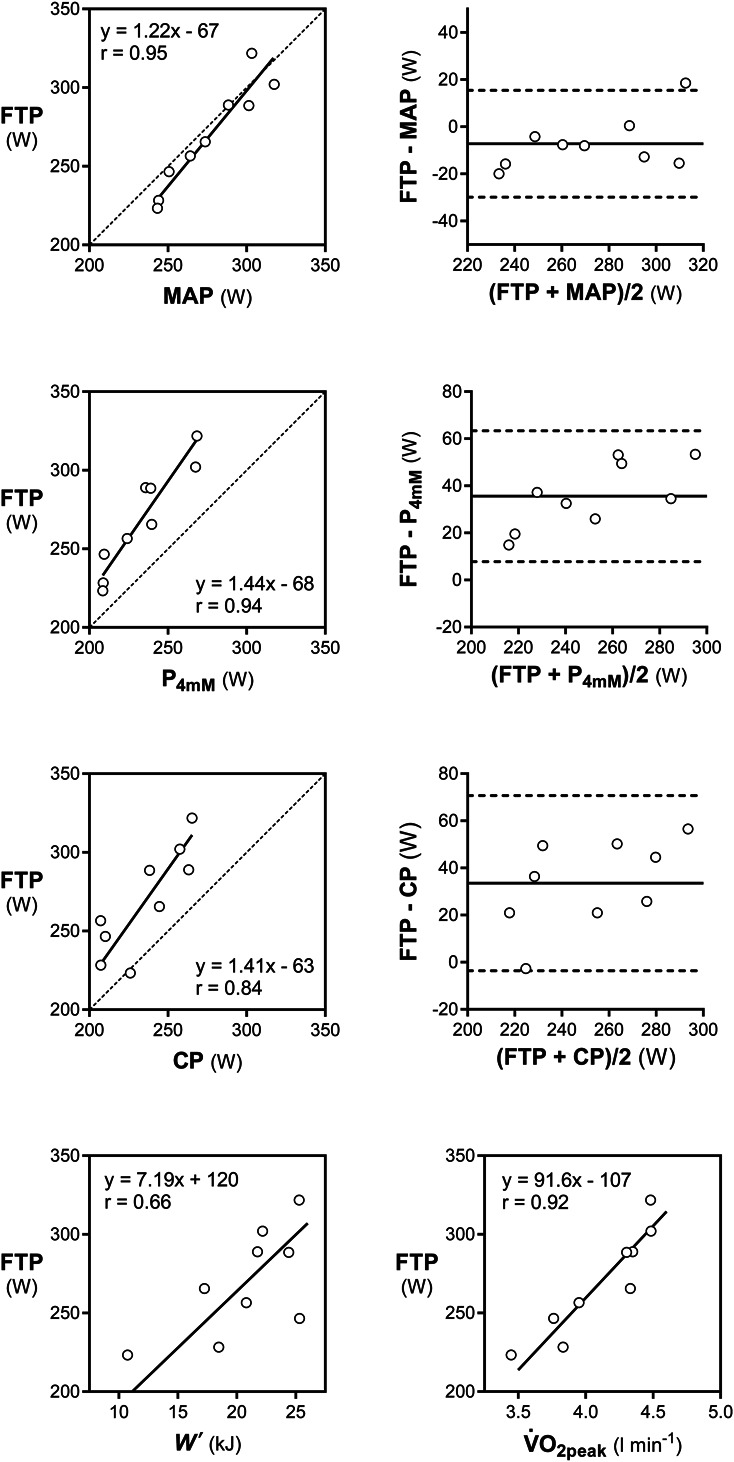
Comparison of selected laboratory parameters with the FTP. FTP = functional threshold power; CP = critical power; P_4mM_ = power output at the 4-mM blood lactate concentration; MAP = maximal aerobic power of the incremental test; $\dot{V}O_{2\text{peak}}$ = peak oxygen consumption of the incremental test; *W*′ = curvature constant of the CP model.

Post–warm-up [La^−^] was 2.0 ± 0.6 mM (*p* = 0.11 vs laboratory). Subjects adopted a positive pacing strategy, with a decrease of −2 ± 1 W·min^−1^ in PO throughout the TT. Mean PO of the TT (283 ± 35 W) significantly outperformed the prediction calculated from the indoor power-duration relationship (*p* < 0.001, *d* = 1.0), and FTP resulted 269 ± 34 W (Figure 2). Physiological measurements obtained during the field 20-minute TT and the incremental test are compared in Table 1. The $\dot{V}O_{2\text{peak}}$ of the 20-minute TT was moderately higher than that of the indoor incremental test (*p* < 0.001, *d* = 1.0). Correlations and agreements are shown in Figure 3. Functional threshold power was significantly higher than CP and P_4mM_ (*p* < 0.001 and *d* = 1.2 for both) although in good correlation with them (*r* = 0.84 and 0.94 respectively). Functional threshold power was not significantly different from MAP (*p* = 0.10, *d* = 0.2) and highly correlated with MAP (*r* = 0.95) and indoor and outdoor $\dot{V}O_{2\text{peak}}$ (*r* = 0.92 and 0.93, respectively). Moreover, FTP was modestly correlated with *W*′ (*r* = 0.66).

**Table 1:** Selected parameters of the FTP field test and the incremental test, whose peak values were used as a reference.*,†

		FTP field test	Incremental test	Effect size (*d*)
Temperature	°C	10.1 ± 2.9‡	20.2 ± 1.2	4.6
Relative humidity		65.2 ± 9.2%‡	52.5% ± 4.6%	1.7
Mechanical power	W	283.2 ± 35.5 (average)	276.3 ± 27.7 (MAP)	0.2
Average cadence	rpm	88.2 ± 5.1‡	99.2 ± 9.3	1.5
Peak HR	bpm	193.4 ± 6.2‡	198.1 ± 5.3	0.9
Average HR	bpm	189.7 ± 6.1	—	
Peak [La^−^]	mM	8.4 ± 1.9‡	11.0 ± 1.7	1.5
Peak $\dot{V}O_{2}$	ml·kg^−1^·min^−1^	66.9 ± 4.4‡	62.7 ± 3.7	1.0
Average $\dot{V}O_{2}$	ml·kg^−1^·min^−1^	63.4 ± 3.7	—	
Gross efficiency		19.0 ± 1.3%	19.0 ± 0.8%	0.0

* FTP = functional threshold power; MAP = maximal aerobic power; HR = heart rate.

† Average HR and oxygen consumption rate ($\dot{V}O_{2}$) were calculated from the last 15 minutes of the FTP field test.

‡ *p* < 0.05 vs. incremental test.

## Discussion

This study was one of the few assessing FTP in field conditions, as well as the very first to involve junior road cyclists and a substantial uphill grade (6.7%). The bioenergetics underlying the 20-minute TT was confirmed mostly aerobic and FTP was closely related, in descending order, with MAP, $\dot{V}O_{2\text{peak}}$, P_4mM_, and CP, as already known for adults (5,16,17,24,27). For the first time, contrarily to previous findings (27), also *W*′ was also found to be moderately related to FTP. This finding is not incompatible with the primary aerobic nature of the 20-minute TT, since, although *W*′ is limited by intramuscular metabolites typical of the anaerobic metabolism (12), it represents the whole energy store available above CP thus including a great aerobic component (9,36). Moreover, the average PO of the 20-minute TT must rely on a complete *W*′ expenditure, thus the correlation with *W*′ remains statistically unaltered if that PO is multiplied by any constant, as 0.95 in the case of FTP.

Strikingly, contrary to laboratory studies in adults, the outdoor 20-minute TT outperformed laboratory tests. The fact that field FTP was essentially equal to laboratory MAP (Figure 3) is to be considered accidental, as MAP can be sustained only for a limited amount of time (although it may not be much above CP in endurance athletes (10,13)). Several theories and facts could be called on to explain this performance increase during the outdoor TT. Motivational issues can be ruled out, as the 20-minute TT was not able to elicit a peak HR or [La^−^] higher than in laboratory conditions, although the HR response may have been attenuated by the lower air temperature in the field by a reduced cardiovascular drift (40). Cycling efficiency was unchanged with respect to the laboratory, in agreement with previous findings regarding high-intensity cycling in uphill and flat terrains (26), despite the higher relative exercise intensity would have suggested a reduction in efficiency due to a higher amplitude of the slow component of the $\dot{V}O_{2}$ kinetics (10,12). Therefore, the increase in performance in the field was possible mainly because of a higher $\dot{V}O_{2\text{peak}}$ coupled with a preserved efficiency. It is known that higher PO may be attained in outdoor vs indoor (25) and in uphill vs flat terrain (29,34), in particular when stationary cycling on an ergometer is compared with free cycling in the field (5,6,25). Recently, it was found that professional cyclists achieved the highest maximal mean PO values at an apparently optimal average gradient of 6–7% (34), strikingly similar to the 6.7% of our 20-minute TT (Figure 1). Riding out of the saddle could also contribute to the increase in short-term PO (26), although our subjects adopted this position only for a handful of seconds across the 20-minute TT. Therefore, high-intensity cycling performed uphill and without restrictions imposed by a stationary trainer is likely to have allowed junior road cyclists to recruit greater muscle mass (e.g., during lateral oscillations), thus enticing a greater $\dot{V}O_{2\text{peak}}$ (9) and its sustainable fraction for 20 minutes. Moreover, the cooler air temperature in the field could have prevented the heat-induced decrease in $\dot{V}O_{2\text{peak}}$ associated with the cardiovascular drift, a phenomenon that likely occurred in the laboratory (40). These findings strengthen the concept that, despite correlations between performance parameters often correlate to each other irrespective of testing conditions, their absolute validity and the derived boundaries between power training zones are specific to those conditions. The effect of unconstrained cycling, uphill grade, and ambient temperature on FTP should be investigated separately in future studies.

In conclusion, in junior road cyclists, the FTP test resulted feasible and related primarily to aerobic endurance parameters and, secondarily but notably, to *W*′. However, absolute performance was significantly higher in outdoor uphill conditions with respect to the laboratory.

**Practical Applications**

In junior road cyclists, FTP was closely related to aerobic endurance parameters and partially also to *W*′. However, outdoor uphill conditions where the FTP test took place were responsible for a significant improvement in PO with respect to laboratory predictions. Specifically, outdoor 20-minute mean PO was 12% higher than that predicted from the indoor power-duration relationship, and the resulting FTP was 14–15% higher than indoor CP and P_4mM_. When translating laboratory results to outdoor training-intensity prescription or performance predictions, coaches and sport scientists should consider these discrepancies, which may be particularly enhanced in this cycling age category.

## Acknowledgments

The authors are grateful to Marco Sbragi of Gobat Srl, Foiano della Chiana, Arezzo, Italy, for his technical assistance. The authors have no conflicts of interest or founding sources to disclose.
